# Effect of host genotype and *Eimeria acervulina* infection on the metabolome of meat-type chickens

**DOI:** 10.1371/journal.pone.0223417

**Published:** 2019-10-16

**Authors:** Samuel E. Aggrey, Marie C. Milfort, Alberta L. Fuller, Jianmin Yuan, Romdhane Rekaya

**Affiliations:** 1 NutriGenomics Laboratory, Department of Poultry Science, University of Georgia, Athens, Georgia, United States of America; 2 State Key Laboratory of Animal Nutrition, College of Animal Science and Technology, China Agricultural University, Beijing, Peoples Republic of China; 3 Department of Animal and Dairy Science, University of Georgia, Athens, Georgia, United States of America; USDA-Agricultural Research Service, UNITED STATES

## Abstract

**Objective:**

A study was conducted to identify metabolic biochemical differences between two chicken genotypes infected with *Eimeria acervulina* and to ascertain the underlying mechanisms for these metabolic alterations and to further delineate genotype-specific effects during merozoite formation and oocyst shedding.

**Methods:**

Fourteen day old chicks of an unimproved (ACRB) and improved (COBB) genotype were orally infected with 2.5 x 10^5^ sporulated *E*. *acervulina* oocysts. At 4 and 6 day-post infection, 5 birds from each treatment group and their controls were bled for serum. Global metabolomic profiles were assessed using ultra performance liquid chromatography/tandem mass spectrometry (metabolon, Inc.,). Statistical analyses were based on analysis of variance to identify which biochemicals differed significantly between experimental groups. Pathway enrichment analysis was conducted to identify significant pathways associated with response to *E*. *acervulina* infection.

**Results:**

A total of 752 metabolites were identified across genotype, treatment and time post infection. Altered fatty acid (FA) metabolism and β-oxidation were identified as dominant metabolic signatures associated with *E*. *acervulina* infection. Key metabolite changes in FA metabolism included stearoylcarnitine, palmitoylcarnitine and linoleoylcarnitine. The infection induced changes in nucleotide metabolism and elicited inflammatory reaction as evidenced by changes in thromboxane B2, 12-HHTrE and itaconate.

**Conclusions:**

Serum metabolome of two chicken genotypes infected with *E*. *acervulina* demonstrated significant changes that were treatment-, time post-infection- and genotype-dependent. Distinct metabolic signatures were identified in fatty acid, nucleotide, inflammation and oxidative stress biochemicals. Significant microbial associated product alterations are likely to be associated with malabsorption of nutrients during infection.

## Introduction

*Eimeria* spp. are apicomplexan parasites that share many metabolic pathways with their animal hosts [1]. The genus *Eimeria* is the largest of the *Eimeriidae* family and is responsible for coccidiosis disease in poultry with global economic losses in excess of $3 billion annually [2]. Upon ingestion by a host, the parasite undergoes a period of asexual reproduction (schizogony) resulting in the production of merozoites which proceeds to a sexual reproduction phase (gametogony) producing macro- and micro-gametocytes. Fertilization of gametocytes is followed by the formation of oocysts which are shed in the feces [3]. *Eimeria* spp. exhibit a high degree of host and site specificity in the gastrointestinal tract, as well as distinct morphology of oocyst and pathogenic effects [4]. The most common parasite species found in chickens are *Eimeria (E*.*) acervulina*, *E*. *maxima* and *E*. *tenella* and they are usually localized in the lower duodenum and upper ileum, mid-ileum near the Meckel’s diverticulum, and caeca, respectively [4,5].

Infection of meat-type chickens (broilers) is often characterized by weight loss, poor feed utilization efficiency, intestinal lesions, diarrhea and ultimately death, depending on the degree of pathogenesis [2,6]. Efficient control of coccidiosis therefore requires rapid and accurate detection and identification of the *Eimeria* spp, however, current diagnosis of infection relies heavily upon post-mortem site specific enteric lesions and observed morphological characteristics during necropsy [4,7,8]. *Eimeria* spp. infection has been identified as one of the predisposing factors of necrotic enteritis in poultry [2–5], therefore control of coccidiosis in poultry is paramount in the control of other enteric disease. Although DNA-based techniques for diagnosis using oocysts in fecal samples have been reported, there are differences in the sensitivity and specificity in detecting different *Eimeria* spp. [9–11].

Progress in omic science has led to significant improvement in the understanding of the molecular and cellular mechanisms that underlie several disease pathologies. Metabolomics is increasingly being used in biomarker discovery, characterization of metabolites and metabolic changes, and development of specific biochemical fingerprints (signatures) for different cellular processes [12,13]. Continuous advances in omics will undoubtedly lead to the development of better diagnostic tools and the discovery of new therapeutics for efficient control of coccidiosis and other diseases in chickens and other species.

Despite the large body of work exploring the metabolome in several species, there has been no comprehensive study of the metabolome of chickens infected with *Eimeria* spp. We hypothesized that infecting different chicken genotypes with *E*. *acervulina* could identify metabolic changes that are likely to be mechanistically involved in the host infection response. We further assessed the metabolomic alterations at two time points (merozoite formation and oocyst shedding) post infection and ascertained genetic background-specific changes and effects.

## Materials and methods

All protocols were approved by the University of Georgia Institutional Animal Care and Use Committee. This research was conducted according to the guidelines approved by the institutional animal care and use committee of the University of Georgia. Two hundred and eighty male chicks comprising of equal numbers of Athens Canadian Random Bred (ACRB) and Cobb500 (COBB) genotypes were raised under standard husbandry practices in coccidian free rooms from hatch until 14 days of age. The ACRB is an unselected control population established in 1955 and has been compared with the modern commercial broiler in several studies. An extensive review of the history and comparative studies involving the ACRB has been previously published [14]. Cobb 500 is a commercial meat-type strain developed by Cobb Vantress Inc., in 1983. Although originally selected for breast meat, the Cobb 500 has been continuously selected for feed efficiency and growth [15]. At 14 days of age, the chicks were randomly assigned to 4 treatments groups in a 2 x 2 factorial design, with 7 replicates per treatment, and 10 birds per replicate. The treatment effects (TX) consisted of bird genotypes (ACRB or COBB) and two infection levels (oral gavage of 2.5 x 10^5^ sporulated *E*. *acervulina* oocysts or distilled water (CTRL)). Birds were fed on a standard non-medicated grower diet, containing 20% crude protein and 12.92 MJ/kg metabolizable energy. Feed and water were provided ad libitum throughout the experiment. Birds were monitored twice a day for any adverse clinical signs. At 4 and 6 days post infection (dpi), 5 birds from each treatment group were randomly sampled and bled according to the University of Georgia animal care protocol. Serum from the blood samples was stored at -86°C. All birds were individually weighed at 0, 4, 6 and 7 dpi.

### Metabolome profiling of chicken serum

#### Sample preparation

The serum metabolomic quantification was performed by Metabolon, Inc. (Durham, NC, USA). The samples were deproteinized by dissociating small molecules bound to protein or trapped in the precipitated protein matrix by precipitating with methanol under vigorous shaking for 2 minutes (Glen Mills GenoGrinder 2000) followed by centrifugation. The resulting extract was divided into five fractions for four different ultra-performance liquid chromatography-tandem mass spectrometry (UPLC-MS/MS) methods: (1) two fractions were analyzed by two separate reverse phase (RP)/UPLC-MS/MS methods with positive ion mode electrospray ionization (ESI); (2) one for analysis by RP/UPLC-MS/MS with negative ion mode ESI; (3) one fraction for analysis by HILIC/UPLC-MS/MS with negative ion mode ESI, and (4) one fraction was reserved for backup. Samples were placed briefly in a TurboVap® (Zymark, Palo Alto, CA, USA) to remove the organic solvent. The sample extracts were stored overnight under nitrogen before preparation for analysis.

#### Ultra-high performance liquid chromatography-tandem mass spectroscopy (UPLC-MS/MS)

For all the methods, a Waters ACQUITY ultra-performance liquid chromatography (UPLC) and a Thermo Scientific Q-Exactive high resolution/accurate mass spectrometer interfaced with a heated electrospray ionization (HESI-II) source and Orbitrap mass analyzer operated at 35,000 mass resolution was used. To ensure injection and chromatography consistency, sample extracts were dried and reconstituted in solvents compatible to each of the four methods. (A): the first aliquot was analyzed using acidic positive ion conditions, chromatographically optimized for more hydrophilic compounds. Using this method, the extract was gradient eluted from a C18 column (Waters UPLC BEH C18-2.1x100 mm, 1.7 μm) using water and methanol, containing 0.05% perfluoropentanoic acid (PFPA) and 0.1% formic acid (FA). (B): the second aliquot was also analyzed using acidic positive ion conditions, however it was chromatographically optimized for more hydrophobic compounds. The extract was gradient eluted from the same aforementioned C18 column using methanol, acetonitrile, water, 0.05% PFPA and 0.01% FA and was operated at an overall higher organic content. (C): the third aliquot was analyzed using basic negative ion optimized conditions with a separate dedicated C18 column. The basic extracts were gradient eluted from the column using methanol and water, however with 6.5mM Ammonium Bicarbonate at pH 8. (D): the fourth aliquot was analyzed via negative ionization following elution from a HILIC column (Waters UPLC BEH Amide 2.1x150 mm, 1.7 μm) using a gradient consisting of water and acetonitrile with 10mM Ammonium Formate, pH 10.8. The MS analysis varied between MS and data-dependent MS^n^ scans using dynamic exclusion. The scan ranged was from 70 to 1000 m/z.

#### Bioinformatics

Raw data files were stored at the Metabolon Laboratory Information Management System (LIMS). The data was extracted and analyzed using peak-identification software. The software has data processing tools for quality control and compound identification, and a collection of information interpretation and visualization tools. Metabolites were identified using Metabolon library which is based on authenticated standards that contain the retention time/index (RI), mass to charge ratio (*m/z)*, and chromatographic data (including MS/MS spectral data) on all molecules present in the library using software developed at Metabolon [16,17]. Over 3,300 commercially available purified standard compounds are registered into LIMS for analysis on all platforms for determination of their analytical characteristics. Area-under-the-curve was used to identify peaks. Whenever necessary, the data was normalized to account for differences in metabolite levels due to differences in the amount of material present in each sample. Data was log transformed and missing values were imputated using the minimum observed value for each compound (S1 Table).

### Statistical analyses

Statistical analysis was based on a model that included the main effects of genotype, treatment and time post infection and their interaction using ArrayStudio (www.omicsoft.com/array-studio). Analysis of variance contrasts were used to identify biochemicals that differed significantly between experimental groups. Statistically significant (p<0.5), as well as those approaching significance (0.05<*p*<0.10) metabolites are reported. An estimate of the false discovery rate (*q*<0.05) was calculated to account for multiple comparisons.

#### Random forest

Random forest (RF) is a supervised ensemble and learning technique used to build predictive models for classification and regression problems [18]. In the current study, RF plots using 30 biochemicals were used to discriminate between genotypes, time post infection and treatment. An accuracy greater than 50% was considered to be better than random chance.

#### Principal components analysis (PCA) and hierarchical clustering analysis (HCA)

PCA analysis was performed using the R package ‘pcaMethods’ with single decomposition [19]. Unsupervised HCA was performed using complete linkage and Euclidian distance where each sample was used as a vector with all the metabolite values to determine sample similarity. Hierarchical clustering analysis was performed by using pheatmap package in R [20]

#### Pathway enrichment analysis

Each contrast was subjected to permutation testing to identify putative pathways containing more significant metabolites than would be expected by random chance alone using proprietary software (Metabolon Inc., Durham, NC, USA). Statistically significant metabolites that were identified were mapped onto general biological pathways using Pathview software package [21].

## Results

### Growth performance

**Fig 1:**
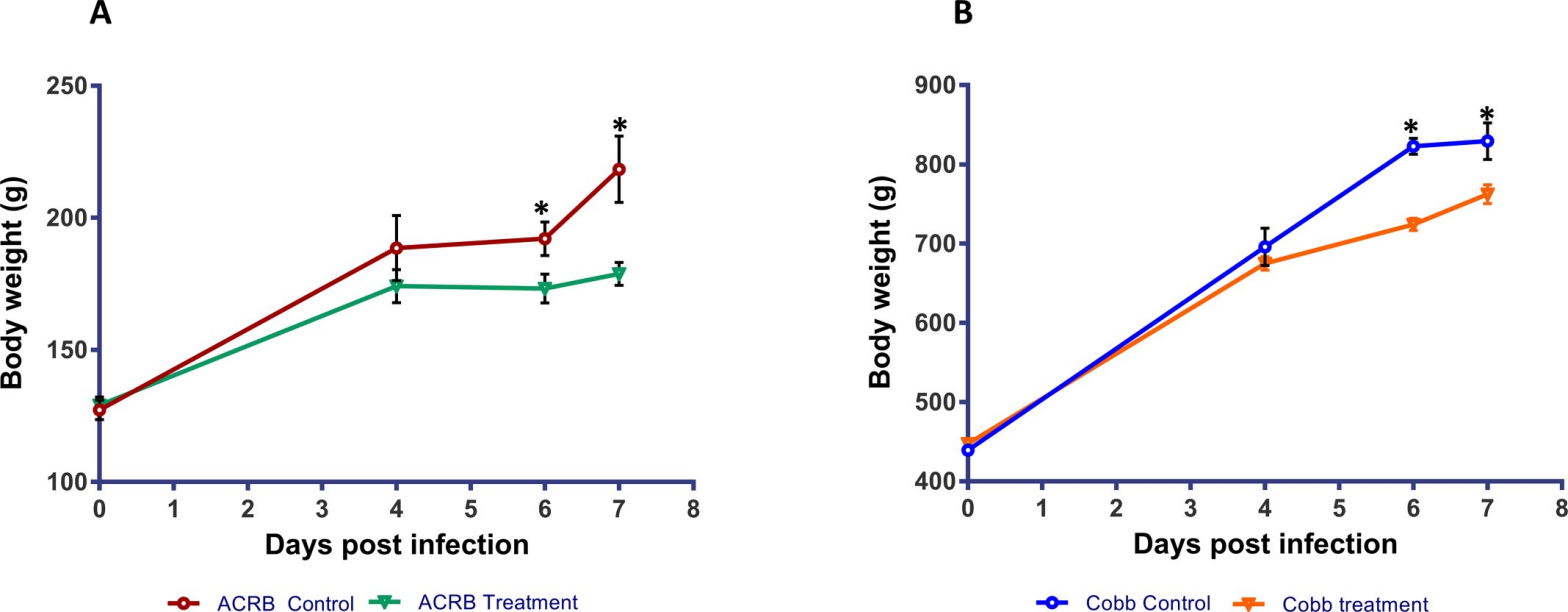
Body weight of chicken genotypes (ACRB and COBB) infected with *Eimeria acervulina* (Treatment) and their uninfected control; *P<0.05. A: represent the Athens-Canadian Random Bred (ACRB) genotype; B: represent the Cobb genotype.

The birds did not show any adverse clinical effects for the duration of the experiment. The growth performance of the ACRB and COBB genotypes are presented (Fig 1).

There were no significant differences in growth among genotypes at 0 and 4 dpi. However, at 6 and 7 dpi, there were significant differences (P<0.05) between the treatment (parasite infection) and control birds within each genotype.

### Metabolome composition

**Fig 2:**
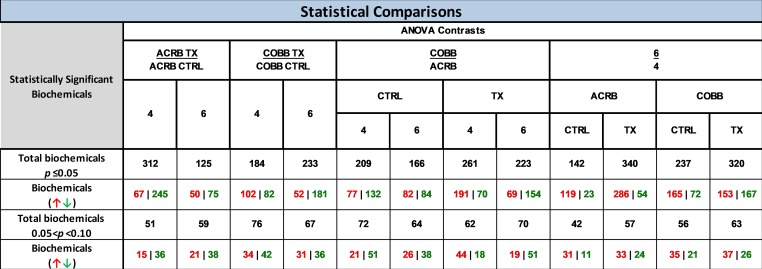
Number of significant metabolite changes by genotype (ACRB and COBB), infection with *Eimeria acervulina* (TX and CTRL), time (4 and 6 days post infection) and their interactions.

A total of 752 metabolites were characterized across genotype, treatment and time post infection classes. The detected metabolites were mapped onto biochemical pathways and grouped into classes by prevalence of lipids (44%), followed by amino acids (27%), xenobiotics (10%), nucleotides (6%), Cofactors/vitamins (4%), carbohydrates (4%), peptides (3%), and energy (2%) (S2 Table). The statistical analysis demonstrates that *E*. *acervulina* infection resulted in the most significant changes among serum samples in the ACRB genotype. In fact, 312 out of the 752 named biochemicals were detected in the ACRB genotype compared to only 184 biochemicals for COBB genotypes at 4 dpi. Additional time point comparisons (6 versus 4 dpi) for samples collected from infected animals revealed 340 significant changes for ACRB and 320 for COBB genotypes indicating similar level of response. Out of the 340 biochemicals that changed in the ACRB treatment group, 286 increased while 54 decreased. In the COBB genotype, 153 biochemicals increased and 167 decreased (Fig 2).

The complete statistical summary of the number of altered biochemicals for the different genotypes, treatment and time post infection are provided (S1 Table).

### Metabolic differences across genotype, infection and time

**Fig 3:**
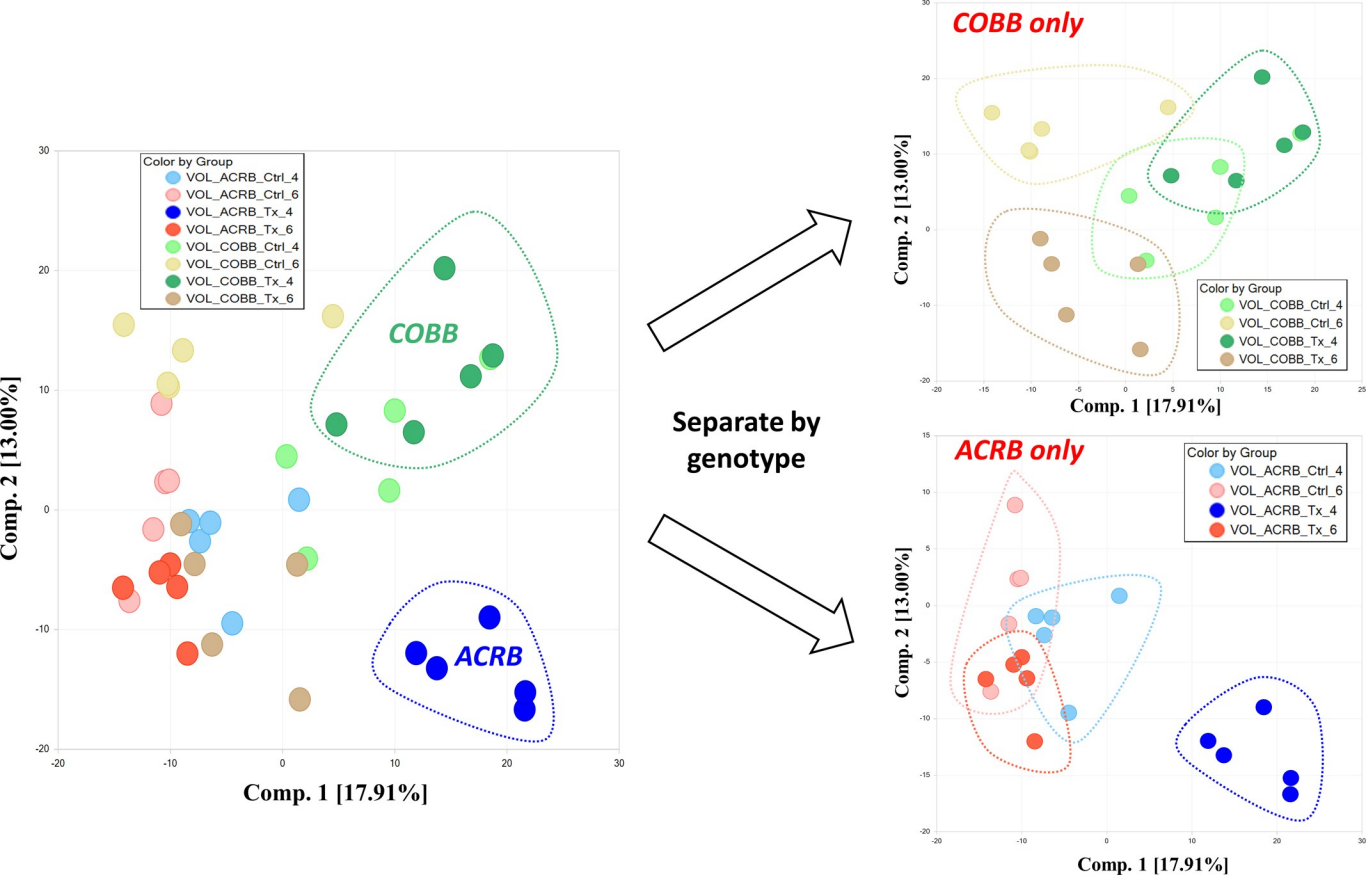
Principal component analysis of metabolic profiles indicating the impact of genotype (ACRB and COBB), infection (TX and CTRL) and time (4 and 6 days post infection with *Eimeria acervulina*). Analyzing the data by genotype shows clustering of genotype and time.

Principal component analysis conducted using all serum samples showed wide distribution with defined separation of infected sera collected 4 dpi from ACRB and COBB genotypes (with partial overlap with 4 day collected control samples) as indicated on Fig 3 (left).

**Fig 4:**
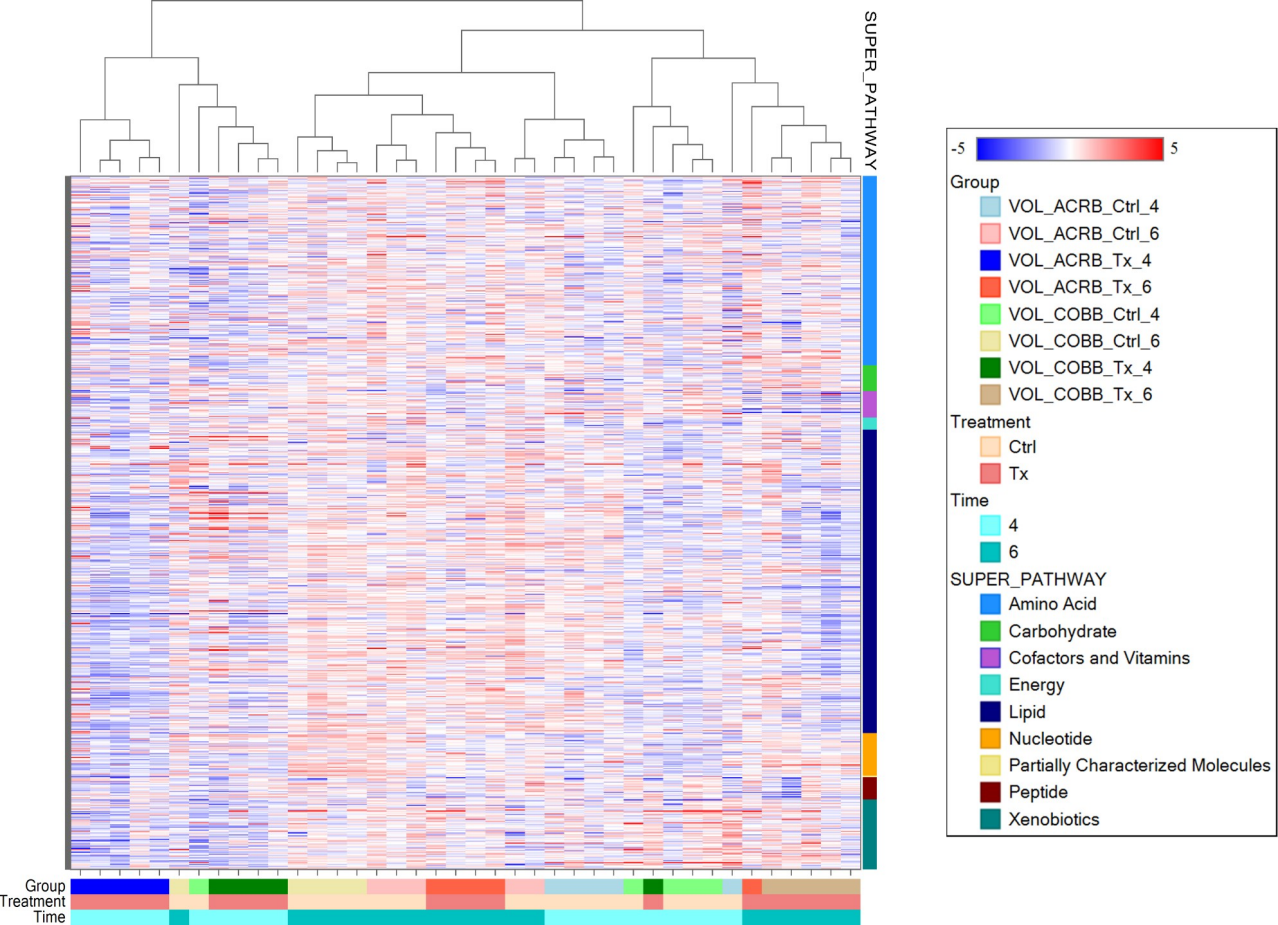
Hierarchical clustering analysis of metabolic profiles indicating the impact of genotype (ACRB and COBB), infection (TX and CTRL) and time (4 and 6 days post infection with *Eimeria acervulina*). Under a given treatment and time combination, red indicates metabolite abundance higher than the mean and blue respresent metabolite abundances lower than the mean. The actual normalized levels of the metabolites were listed in S1 Table.

Surprisingly, sera collected at 6 dpi from inoculated birds was similar to the controls. Additional assessment restricting the analysis to samples collected either from ACRB (Fig 3, right bottom) or COBB (Fig 3, right top) genotype demonstrate better separation for 6 dpi Cobb collected samples (infected versus control) and 4 dpi ACRB serum samples. The hierarchical clustering analysis based on stepwise clustering method grouped metabolically similar samples close to each other, and, in agreement with PCA, showed limited sub-clustering based on time post infection and treatment (Fig 4).

**Fig 5:**
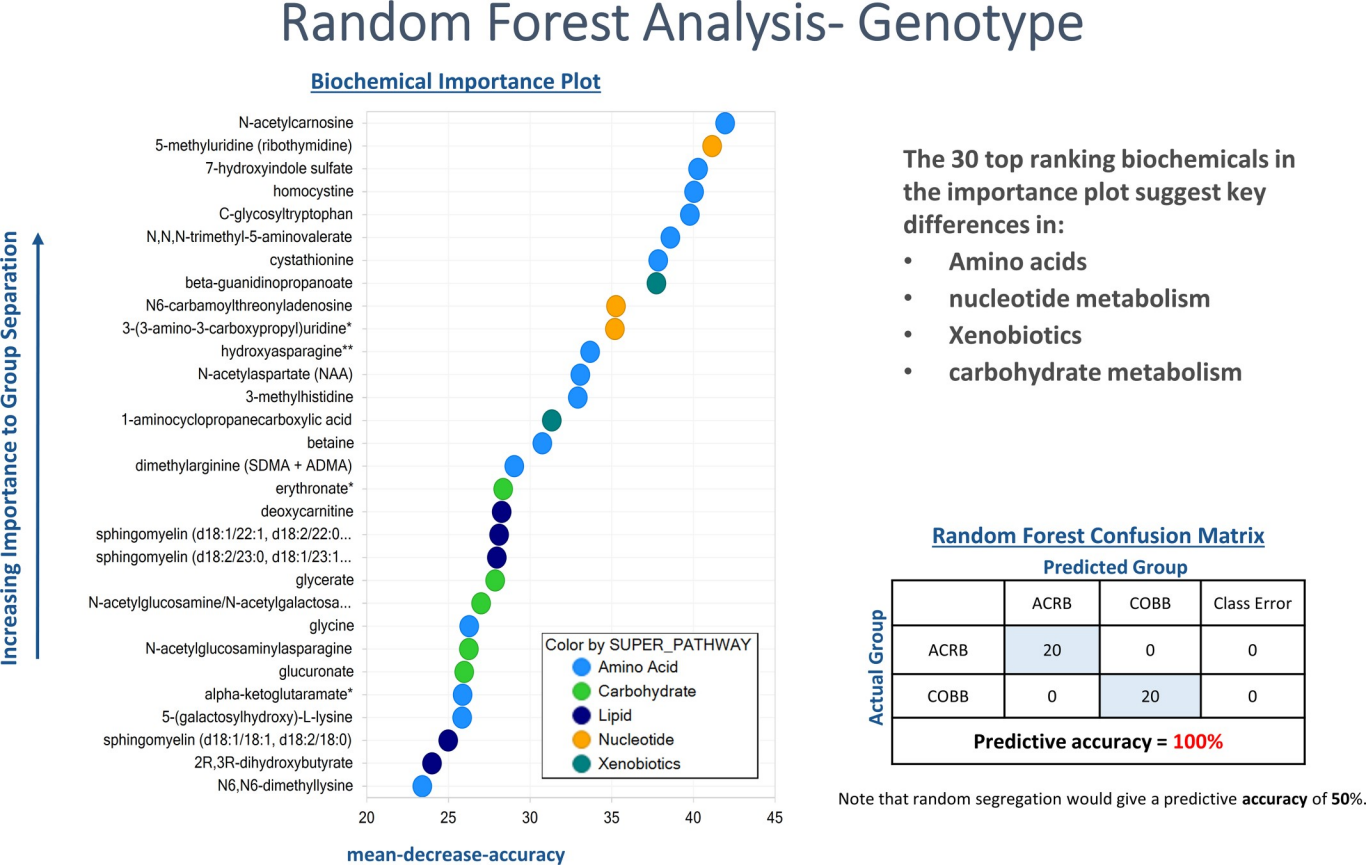
Random forest plot of top 30 biochemical present in serum samples of chicken genotypes (ACRB and COBB) infected with *Eimeria acervulina*. Compounds contributing the most to the accuracy of disease classification were identified as those having the highest mean decrease accuracy (MDA). A higher MDA value indicates a greater predictive value.

The top-level split separates samples collected at 4 dpi from other samples. Secondary split by genotype was also present, with time post infection as a confounding variable because there were untreated (control) and infected groups. The RF classification uniquely identified biomarkers differentiating classification groups. RF analysis using biochemical data derived from ACRB and COBB chickens resulted in predictive accuracy of 100% compared to 50% expected by random chance alone (Fig 5).

**Fig 6:**
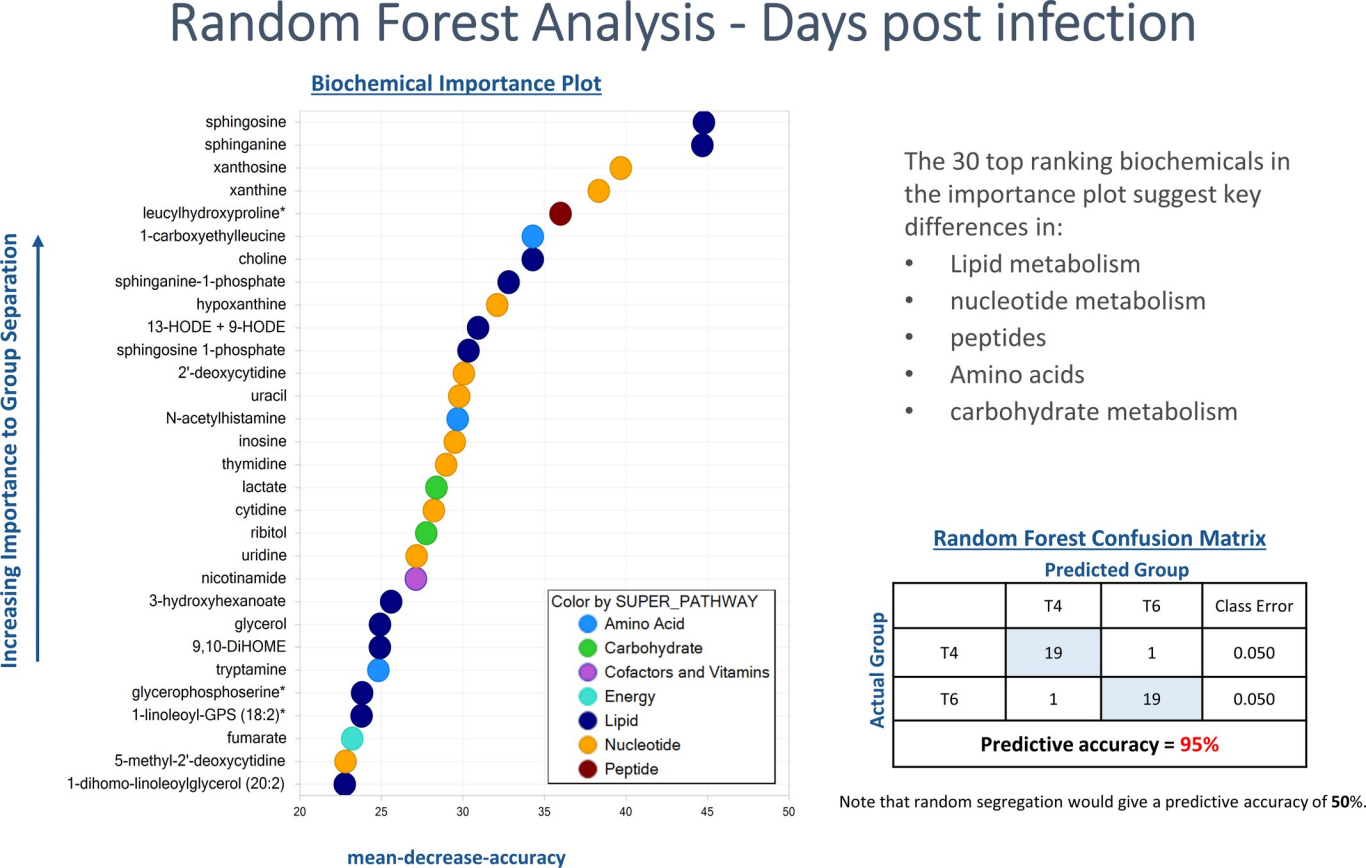
Random forest plot of top 30 biochemical present in serum samples of chickens infected with *Eimeria acervulina* for 4 (T4) and 6 (T6) days. Compounds contributing the most to the accuracy of disease classification were identified as those having the highest mean decrease accuracy (MDA). A higher MDA value indicates a greater predictive value.

RF analysis also generates an accompanying list of the top metabolites contributing most to the separation of the genotypes. The top 30 metabolites separating ACRB and COBB genotypes pointed heavily to amino acids, nucleotides, xenobiotics and carbohydrates. The same analysis using biochemicals identified from samples collected at 4 and 6 dpi resulted in predictive accuracy of 95% (Fig 6), with the top discriminating metabolites pointing towards lipid metabolism (e.g. 13-HODE+9-HODE), nucleotides, peptides and amino acids.

**Fig 7:**
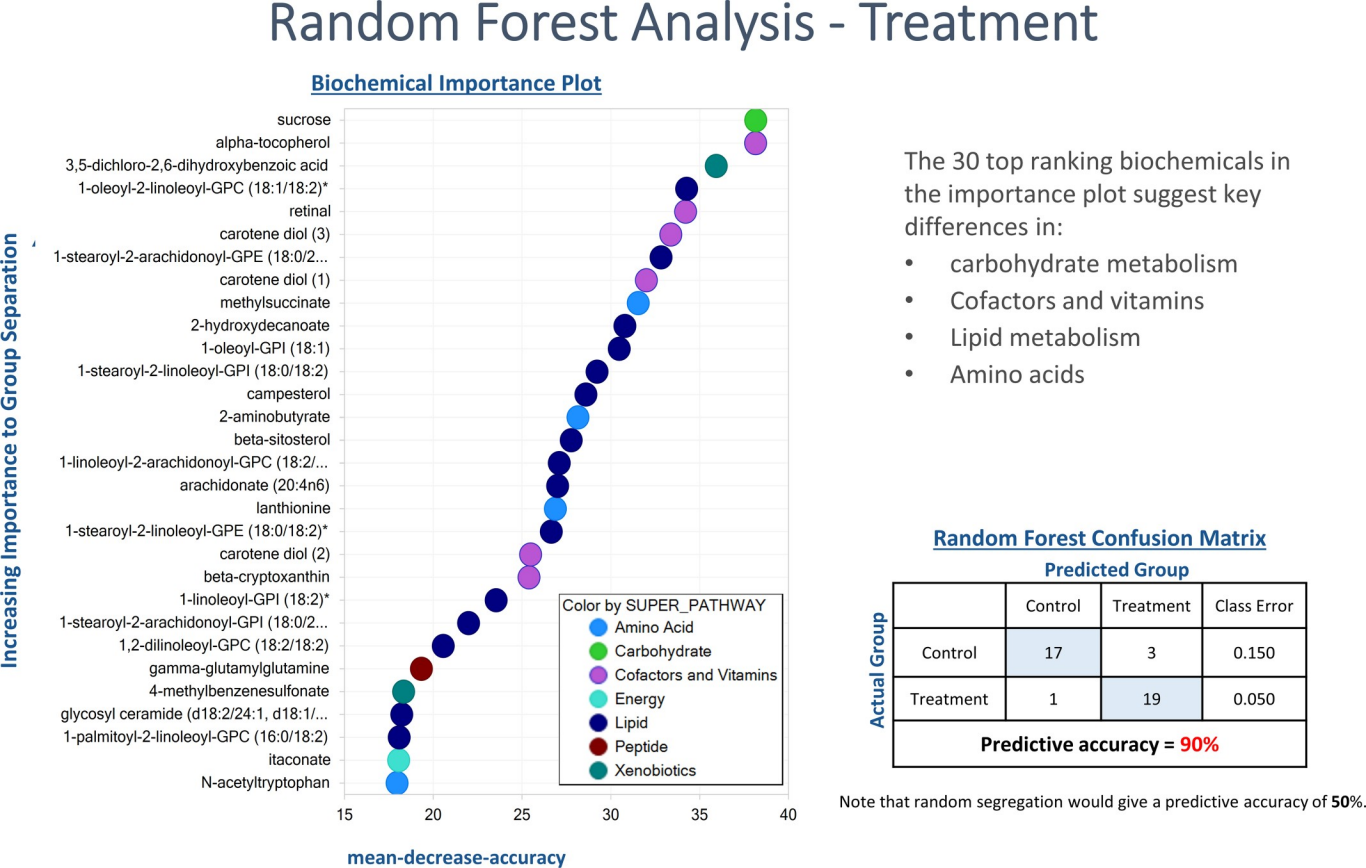
Random forest plot of top 30 biochemical present in serum samples of chicken infected with *Eimeria acervulina* (Treatment) and their control. Mean decrease was used as a measure of variable importance. Compounds contributing the most to the accuracy of disease classification were identified as those having the highest mean decrease accuracy (MDA). A higher MDA value indicates a greater predictive value.

Additionally, RF analysis using samples collected from control and infected animals resulted in predictive accuracy of 90% (Fig 7) with the main discriminating metabolites including carbohydrates, cofactors and vitamins and lipid metabolism.

### Changes in lipid metabolism due to *E*. *acervulina* infection

**Fig 8:**
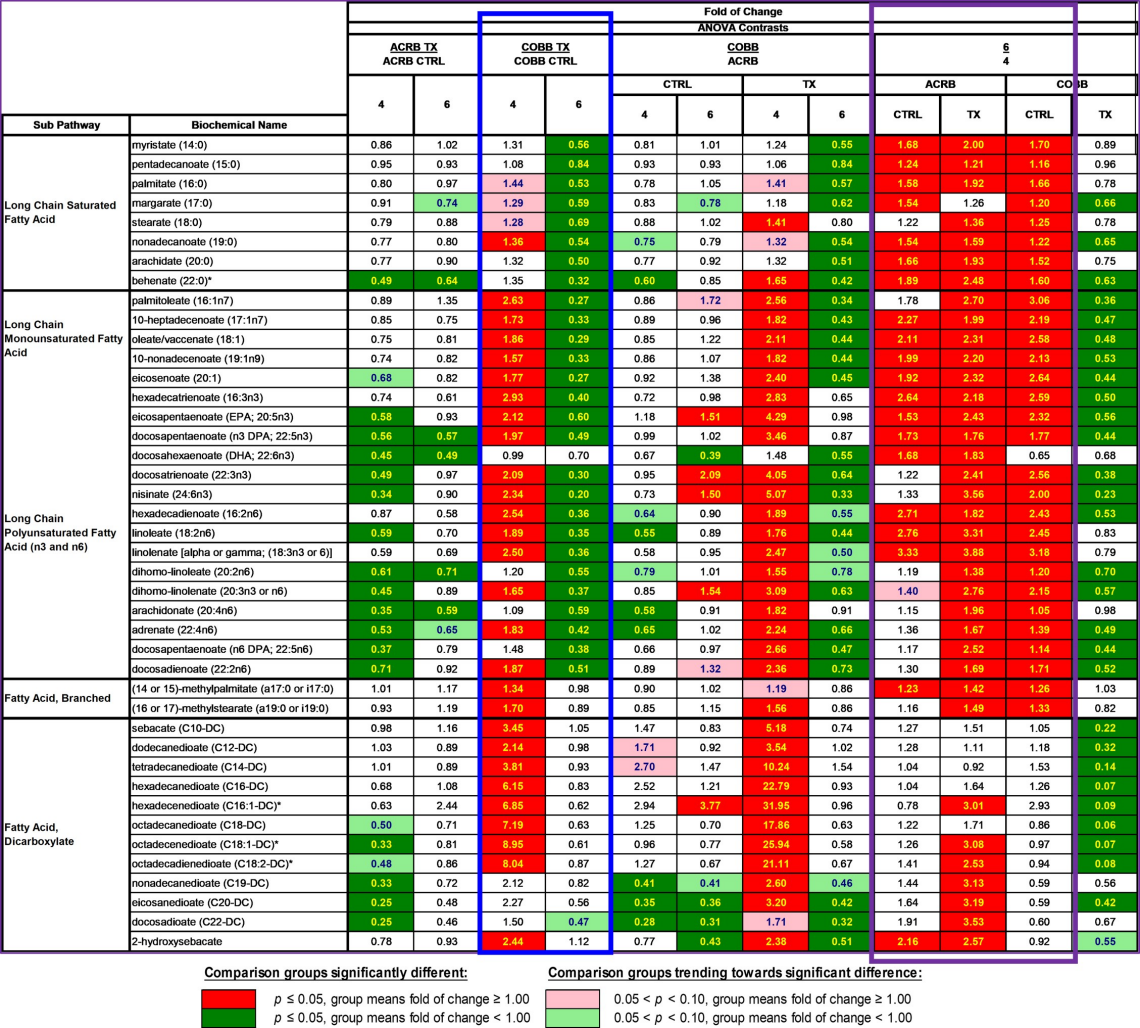
Changes in fatty acid metabolism in chicken genotypes (ACRB and COBB) infected (TX and CTRL) with *Eimeria acervulina* and their interactions at 4 and 6 days post infection.

A metabolic signature of altered fatty acid mobilization and β-oxidation was observed as a function of infection status (*E*. *acervulina* infection vs control) and with time post infection (6 vs 4 dpi) as evidenced by significantly elevated levels of long chain-, monosaturated-, polyunsaturated, and dicarboxylate- fatty acids in serum samples collected 6 versus 4 dpi in the ACRB (infected and controls) and COBB (control only) (blue box, Fig 8), suggesting a potential interaction between time post infection and treatment for these compounds. The COBB genotype responded with significant changes in lipid utilization due to *E*. *acervulina* infection, where levels of most of the long chain and dicarboxylate fatty acids were significantly elevated at 4 dpi and decreased 6 dpi post infection (purple box, Fig 8).

**Fig 9:**
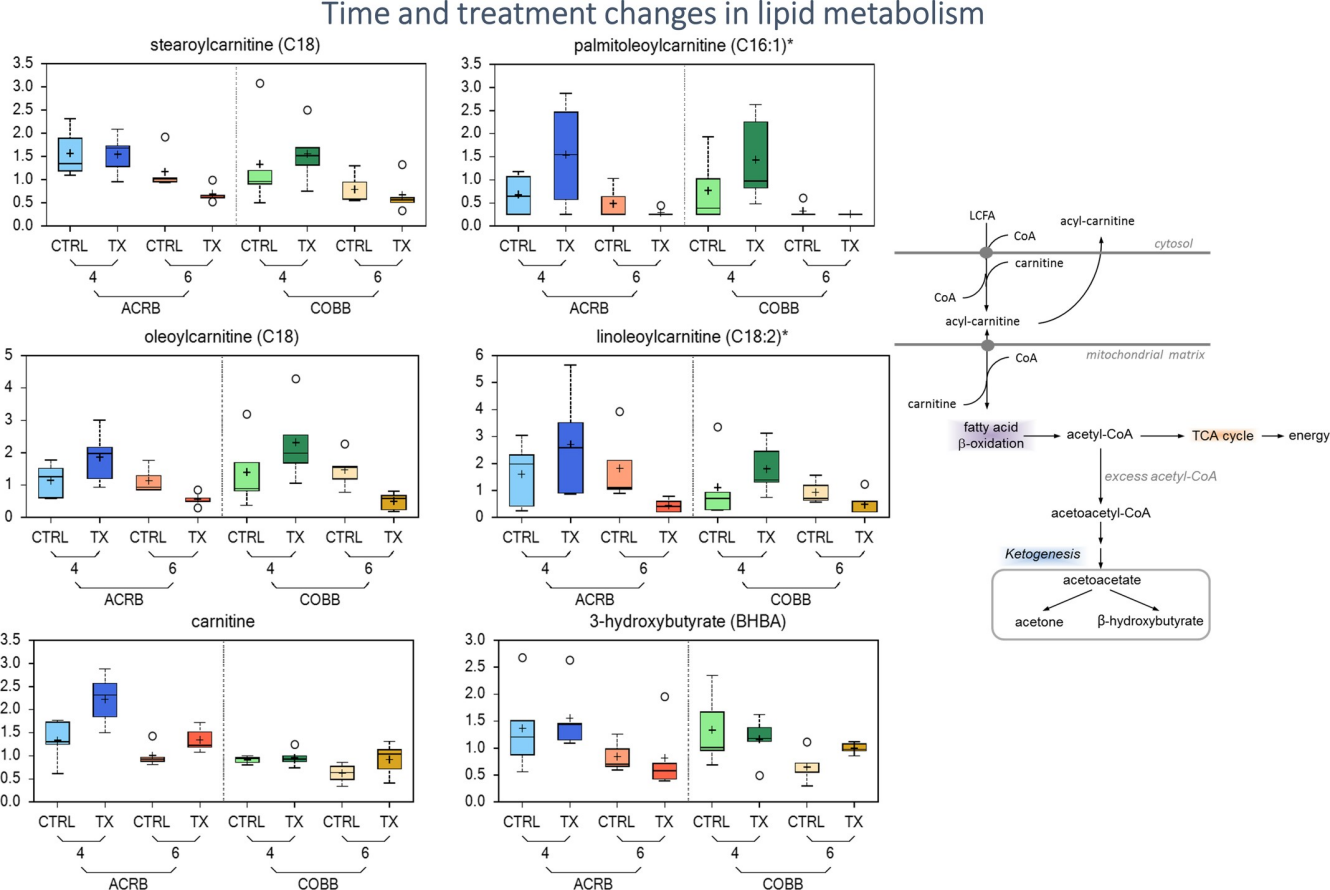
Differences in abundance of lipid metabolites in chicken genotypes (ACRB and COBB) infected with *Eimeria acervulina* (TX) and their control (CTRL) at 4 and 6 days post infection. The adjusted P-values for significant comparison are presented in S2 Table. The upper whiskers represent the maximum, and the lower whiskers the minimum values. The plus-signs indicate the mean values, while the median values are represented by the black line within the boxes.

There were significant decreases in lysophospholipids (2-arachidonolyl-GPA (20:4), 1-palmitoyl-GPE (16:0), 1-stearoyl-GPE (18:0)) in the COBB treatment group compared with the controls at 4 dpi (S2 Table). Additional changes in the levels of metabolites associated with fatty acid β-oxidation, including carnitine conjugated fatty acids such as stearoylcarnitine (C18), palmitoylcarnitine (C16), oleoylcarnitine (C18:1) and linoleoylcarnitine (C18:2)* along with ketone body 3-hydroxybutyrate (Fig 9) is suggestive of alterations in serum free fatty acid (FFA) utilization, and β-oxidation as a response to *E*. *acervulina* infection (in relation to control) and time post infection (6 vs 4 dpi).

### Infection related changes in inflammation and oxidative stress

**Fig 10:**
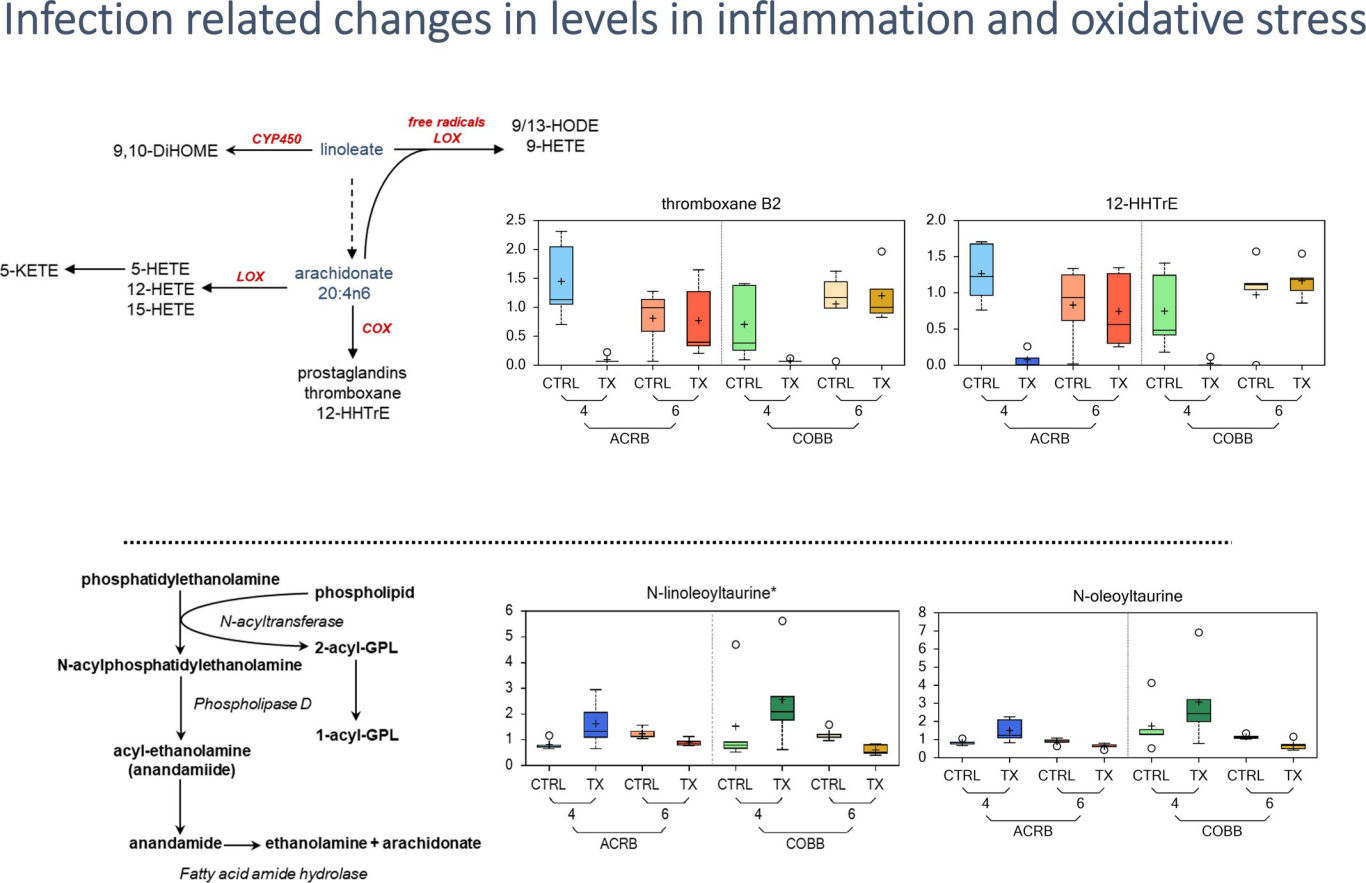
Differences in abundance of inflammation and oxidative stress metabolites in chicken genotypes (ACRB and COBB) infected with *Eimeria acervulina* (TX) and their control (CTRL) at 4 and 6 days post infection. The adjusted P-values for significant comparison are presented in S2 Table. The upper whiskers represent the maximum, and the lower whiskers the minimum values. The plus-signs indicate the mean values, while the median values are represented by the black line within the boxes.

Inflammation is a highly regulated process with multiple inputs, including oxidized lipids (eicosanoids, the products of LOX/COX activation) and other regulatory compounds. Infection led to a strong decrease in the eicosanoids, thromboxane B2 and 12-HHTrE for both genotypes at 4 dpi and recovered by 6 dpi (Fig 10).

This reduction was more pronounced in the ACRB strain. Finally, in contrast to the decline in eicosanoids, increases in itaconate (an inhibitor to bacterial growth) were observed at both 4 and 6 dpi.

### Nucleotide metabolism

**Fig 11:**
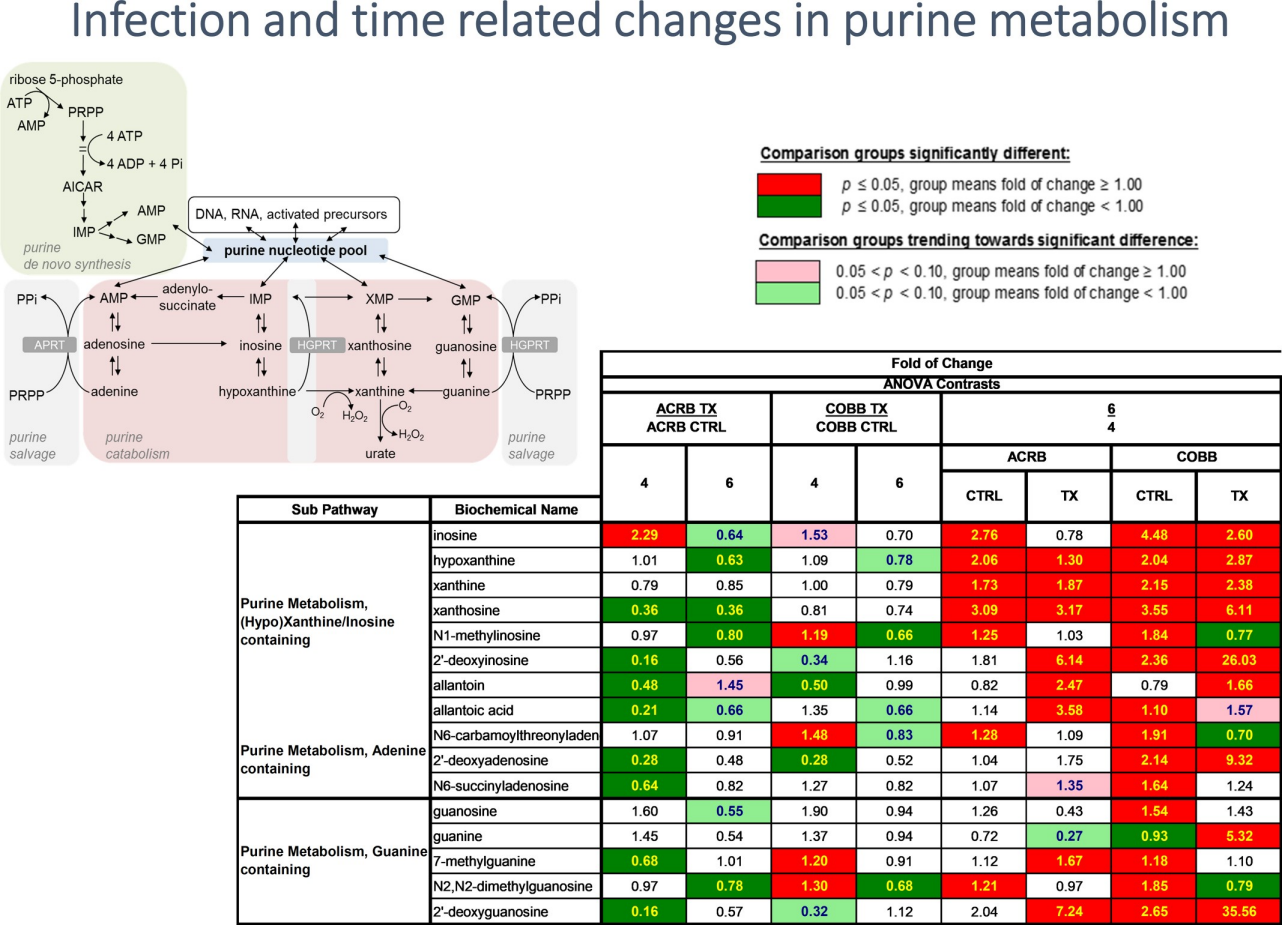
Differences in abundance in serum nucleoties in chicken genotypes (ACRB and COBB) infected with *Eimeria acervulina* (TX) and their control (CTRL) at 4 and 6 days post infection. The adjusted P-values for significant comparison are presented in S2 Table.

Based on the hierarchical clustering presented in Fig 4, it is reasonable to infer that the time post infection (6 vs 4 dpi) had a dramatic impact on the level of several metabolites reporting on these processes with the significant increase in most of the detected purine (inosine, xanthine, guanine, 2’deoxyguanosine) and pyrimidine (uracil, cytosine, thymidine and thymine) metabolites (S2 Table). In particular, infection with *E*. *acervulina* led to a decline in purine metabolism in the ACRB genotype. However, the level of sera nucleotides increased from 4 to 6 dpi (Fig 11).

## Discussion

In the current study, an untargeted serum metabolome of two chicken genotypes infected with *E*. *acervulina* was performed at 4 and 6 dpi to characterize the joint biochemical and metabolic changes that occurred during the infection. We provide the first mechanistic insight into the metabolic alterations that occur after *E*. *acervulina* infection during merozoite formation (4 dpi) and oocyst shedding (6 dpi) in ACRB and COBB genotypes. The Cobb 500 has been used in other studies involving *E*. *acervulina* [22–24] and the ACRB was used to study the genetic variability to *E*. *acervulina* and *E*. *tenella* [25]. Data on comparative analysis of the responses of the Cobb and ACRB genotypes to *Eimeria* spp is scant.

The PCA analysis by genotype pointed towards a differential time response. The ACRB genotype was developed in the 1950s and has been maintained as an unselected control population [14], whereas the COBB genotype is a commercial strain under constant genetic improvement. Even though the body weights of both genotypes at the time of infection were significantly different (Fig 1), they were both infected with the same dose of *E*. *acervulina* sporulated oocysts. Thus, potential parasitic load could be responsible for the differential time response as indicated by the PCA analysis. Infecting the two genotypes at similar body weight to ensure equivalent parasitic load could be done, however, the genotypes would be at significantly different ages which could introduce other sources of variation. The differential amino acids, nucleotide, xenobiotics and carbohydrate metabolism (Fig 5) as predicted by the RF analysis could also contribute towards the differential time response of these two genotypes.

The main biochemical changes between infected and control birds are differentiated by carbohydrates, cofactors, vitamins and lipid metabolism as indicated by the RF analysis (Fig 6). In the current study, the serum glucose levels were unchanged between infected and uninfected groups regardless of the genotype (S2 Table). This is not surprising given the known ability of chickens to maintain a stable blood glucose level under a variety of stressors [26]. Sucrose levels significantly changed in the infected birds. Monosaccharide transporters, e.g. GLUT2, GLUT5 and SGLT1 have been shown to be downregulated in the gut epithelia during *E*. *acervulina* infection [27]. However, the mRNA expression of the putative animal sucrose transporter (SLC45) [28] in chickens remains unknown. Other biochemicals that changed in the infected group compared to the controls included alpha-tocopherol, retinal, carotene diol, 2-aminobutyrate, itaconate and N-acetyltryptophan. Chickens infected with *E*. *acervulina*, which primarily affects the lower duodenum and jejunal mucosas [29], suffer nutrient malabsorption including that of carotenoids [30,31]. The physical damage to the absorptive mucosa could be responsible for the reduced absorption of vitamins A, E, carotenoids [27,31,32] and other cofactors leading to their concomitant reductions in the serum (S2 Table).

The physical disruption to the intestinal mucosa villi and epithelium [33] and alterations in lipid transport within mucosal epithelial cells [34] have been previously associated with lipid malabsorption. Young chickens infected with *E*. *acervulina* have been shown to have a reduction in the pH of the intestinal content [35] which may cause an impairment of lipid hydrolysis [36]. According to Webb et al. [37], increased levels of unabsorbed lipids may cause diarrhea due to bacterial production of C-18 fatty acids. The dynamics of fatty acid metabolism depended on the genotype. In the ACRB birds, there was a significant reduction in long chain monosaturated fatty acids (LCMFA) at 4 dpi, but the opposite was observed in the COBB genotype. There were significant increases in LCMFA, long chain polyunsaturated fatty acid and fatty acid dicarboxylate levels in the COBB infected birds at 4 dpi compared to the ACRB birds, however, between 4 and 6 dpi, the COBB infected chickens showed a reduction in LCMFA, long chain polyunsaturated fatty acids and fatty acid dicarboxylate pointing to a better fatty acid absorption in the COBB genotype compared to their ACRB counterparts. These carnitine-conjugated fatty acids are transferred from the cytosol to the mitochondrial matrix to undergo β-oxidation (Fig 9), supplying acetyl-CoA to the TCA cycle to generate energy. Induction of β-oxidation to generate energy during *E*. *acervulina* infection could be a mechanism to compensate for the reduced energy and protein retentions [34]. Further, ketone body 3-hydroxybutyrate can regulate cellular processes directly through the inhibition of histone deacetylase, binding to cell surface receptors and by altering acetyl-CoA, succinyl-CoA, and NAD+ [38]. Previous research on the effect of *E*. *acervulina* infection on lipid metabolism have also shown a significant reduction in high-density lipoproteins, low-density lipoproteins, and very low-density lipoproteins in infected chickens [39,40] which were all attributed to nutrient malabsorption and liver changes. Reduced proteins are associated with deficiency in lipotropic factors which are important for the synthesis of phospholipids and lipoproteins [41]. Disruption of the epithelia of lower jejunum and upper ileum by *E*. *acervulina* and its negative effect on absorption of nutrients may be a major factor contributing to the decrease in growth of infected chickens (Fig 1).

Cyclooxygenase products thromboxane B2 (TXB2) and 12-Hydroxyheptadecatrienoic acid (12-HHTrE), products of inflammation sites [42,43], decreased in both genotypes and infection groups at 4 dpi. However, both TXB2 and 12-HHTrE serum levels significantly increased at 6 dpi suggesting an early repression of inflammation signaling with infection. This reduction was strongest in the ACRB genotype. Finally, in contrast to the decline in eicosanoids, increases in itaconate (S2 Table) were observed at both 4 and 6 dpi. Itaconate is an anti-inflammatory metabolite that regulates macrophage function and is required for the activation of anti-inflammatory transcription factor Nrf2 [44–46]. Taken together, the changes in the aforementioned metabolites suggest a potential modulation of inflammation by the host in response to *Eimeria*.

Endocannabinoids, a group of endogenous bioactive lipids possess immunomodulatory effects and are frequently associated with inflammation and pain [47], often serving as a brake on inflammation via cannabinoid or vanilloid receptors [48–50]. The levels of those biochemicals were increased in treatment versus control sera 4 dpi of the COBB genotype consistent with potential activation of immune system when responding to infection, however, at 6 dpi, when the infected birds were compared with their control counterparts, levels of all detected endocannabinoids were decreased (oleoyl ethanolamide, N-linoleoyltaurine*, linoleoyl ethanolamide and N-oleoylserine) reaching statistical significance (S2 Table). Furthermore, significant increases in levels of kynurenine (6 vs 4 dpi) were observed for both genotypes (Fig 10). Since the formation of kynurenine through ubiquitous indoleamine-2,3-dioxygenase (IDO) is stimulated by pro-inflammatory cytokines, kynurenine may serve as a biomarker of inflammation [51,52]. Overall, serum samples collected post infection showed significant changes in markers of inflammation and oxidative stress that were altered based on time of collection and genotype of the bird. It should be pointed out that serum biomarkers reflect global metabolism and may be impacted by induction of factors limiting inflammatory mediators (in the liver, for example, which processes blood draining from the gut).

During normal growth, nucleic acids are constantly being degraded and resynthesized. Purine and pyrimidine nucleotides can be broken down into nucleosides and nucleobases. These nucleosides can be broken down further or recycled back into nucleotides via salvage pathways [53]. Comparing 4 and 6 dpi, it can be inferred that *E*. *acervulina* infection had a dramatic impact on the levels of several metabolites reporting on these processes with the significant increase in most of the detected purine and pyrimidine metabolites (S2 Table). This unidirectional change is suggestive of a potential increase in DNA/RNA turnover and utilization of these nucleotides [54]. Purine degradation is regulated, in part, by oxidative stress, and it is linked to inflammation [55,56] which could be reflective of the response to *E*. *acervulina* infection.

**Fig 12:**
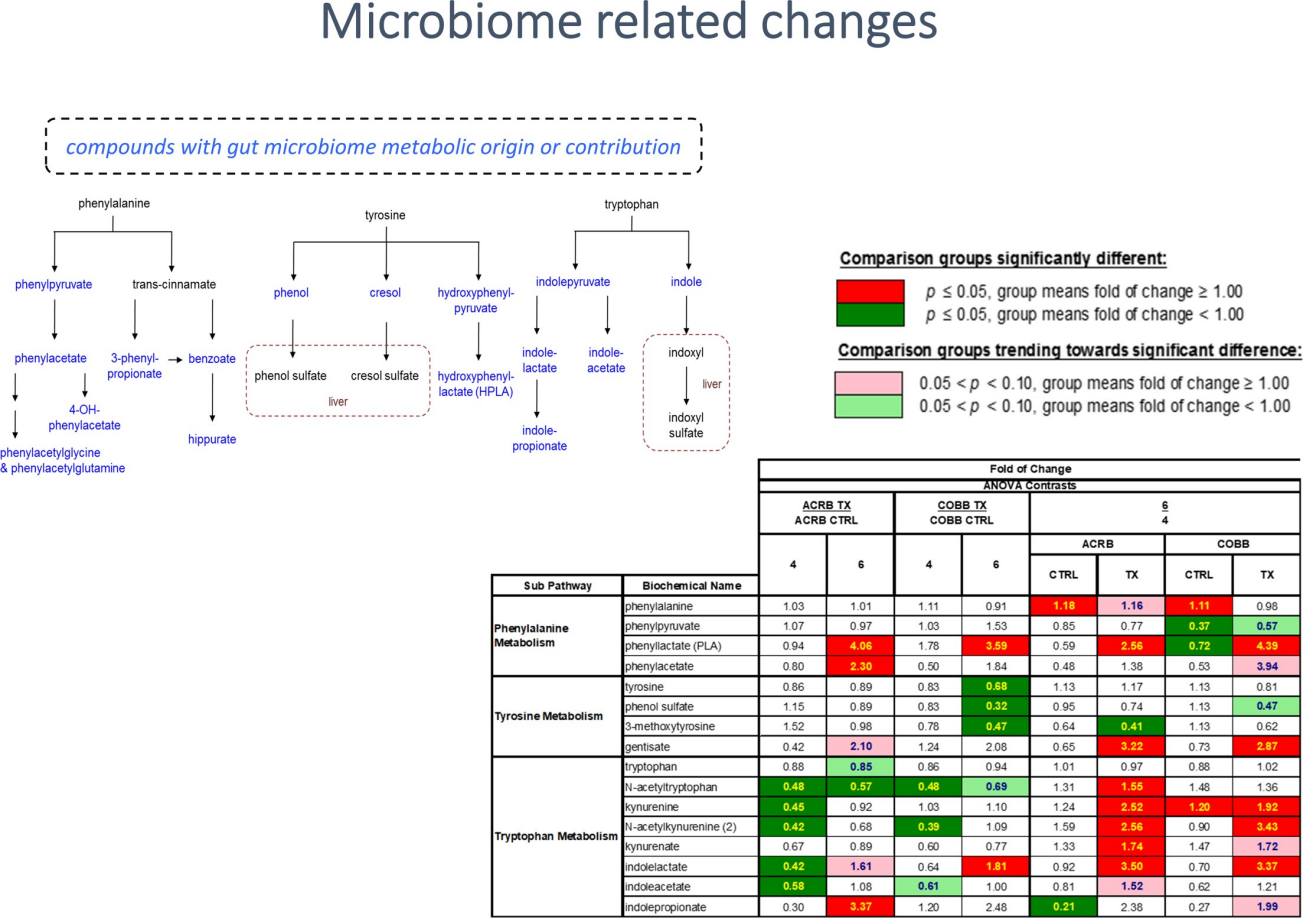
Compounds with microbial metabolism origin in chicken genotypes (ACRB and COBB) infected with *Eimeria acervulina* (TX) and their control (CTRL) at 4 and 6 days post infection. The adjusted P-values for significant comparison are presented in S2 Table.

There were changes in microbiome-associated products of histidine (imidazole lactate and imidazole propionate), phenylalanine (phenylpyruvate, phenylacetate and phenyllactate (PLA), tyrosine (phenol sulfate) and tryptophan (indolelactate, indoleacetate and indolepropionate) metabolism (Fig 12), which could reflect infection associated changes in the gut microbiome, where most of the biochemicals are elevated in samples collected at day 6 vs 4 dpi.

The gastrointestinal tract of poultry is densely populated with microorganisms which closely and intensively interact with the host and ingested feed. In the current study, several gut microbiome derived metabolites were altered by infection as well as the time post infection, suggesting changes in the composition of microbiota. The metabolism of aromatic amino acids, phenylalanine, tryptophan and tyrosine occurs, in part, through the involvement of enzymes encoded within the microbiome. Collectively, the altered fatty acid signature may be attributable to alterations in the metabolic program of the intestinal microbiome contributing to differential lipid handling. Soluble dietary fibers and resistant starch can be fermented by gut microbiota, producing short chain fatty acids (SCFAs), namely acetate, propionate, and butyrate. In this study, levels of butyrate were significantly decreased due to infection in ACRB chickens compared to control samples collected at 6 dpi, further confirming infection related changes in gut microbiota. Diet supplementation with nutriceuticals, prebiotics and probiotics and lipids aimed at ameliorating some of the effects of malabsorption caused by *E*. *acervulina* could reduce potential dysbiosis and contraction of other enteric diseases.

## Conclusion

The global metabolome of chicken serum samples from two genotypes (ACRB and COBB) that were infected with *E*. *acervulina* and collected 4 and 6 dpi demonstrated a significant number of changes that were treatment, time post infection and genotype dependent. In-depth analysis showed significant alterations in fatty acid metabolism that might be associated with changes in lipid absorption and utilization. The time course comparison showed significant changes in markers of inflammation and oxidative stress illustrating changes in response to infection. Changes in microbiome-associated products of histidine, phenylalanine, tyrosine and tryptophan metabolism were observed, which could reflect infection associated changes in the gut microbiome. Global profiling of intestinal tissues and contents would provide additional insight into the metabolic and microbiome effects associated with *E*. *acervulina* infection. The intriguing changes in inflammatory metabolites at 4 and 6 dpi invite data collection beyond 6 dip which could lead to the identification of additional factors associated with host response to *E*. *acervulina*.

## Supporting information

S1 Table Individual metabolite data. Dataset used for analysis.
(XLSX)

S2 Table Metabolites that differ post infection in chicken genotypes. Metabolites with fold change and the level of significance by genotype, time and genotype by time interaction.
(XLSX)
